# Determinants of career aspirations of medical students in southern China

**DOI:** 10.1186/1472-6920-8-59

**Published:** 2008-12-11

**Authors:** LingBing She, BingLi Wu, LiYan Xu, JianYi Wu, PiXian Zhang, EnMin Li

**Affiliations:** 1 Department of Biochemistry and Molecular Biology, Shantou University Medical College, Shantou, PR China; 2 Department of Pathology, the Key Immunopathology Laboratory of Guangdong Province, Shantou University Medical College, Shantou, PR China

## Abstract

**Background:**

With recent changes in both the Chinese medical system and compensation of medical doctors, the career aspirations of Chinese medical students have become more diverse. Shantou University Medical College has conducted evaluations and instituted programs to enhance student preparedness to enter a variety of medical careers.

**Methods:**

A survey was conducted with 85 students to evaluate medical career aspirations and their association with family background, personal skills, English language proficiency, and interest in biomedical research, which were considered as possible factors affecting their career interest.

**Results:**

Chinese students aspire to traditional as well as nontraditional medical careers. A significant minority of students are now interested in nontraditional careers such as medical teaching or research. However, poor proficiency in the English language and lack of computer skills may limit their academic and career opportunities.

**Conclusion:**

Career aspirations have changed among medical undergraduates. Although many wish to pursue a traditional clinical doctor career, many are interested in research and teaching careers. Factors such as family background, personal characteristics, school mentoring, and extracurricular support may play a role.

## Background

The traditional view held by Chinese university medical students is that their career path is limited to clinical medicine following graduation [1]. However, there is a growing trend toward nonclinical career aspirations among current medical students [2]. A possible contributing factor is the conflict between increased medical student recruitment and the intensive competition for positions in prosperous cities. Students seeking to practice in major cities rather than in rural locales may face great employment pressure in the near future. Development of students' research skills while in medical school may increase the competitiveness of graduates for future employment options [3]. Previous studies have shown significant changes in intended career choice following participation in such programs [4,5]. Shantou University Medical College (SUMC), which offers 5-year and 7-year training programs for clinical medicine, has grown rapidly thanks to the generous financial support of the Li Ka Shing Foundation of Hong Kong. Faculty members at SUMC believe that exposing undergraduate medical students to research early in their education is crucial to the students' career development. Recently, the Department of Biochemistry and Molecular Biology at SUMC created the "Outstanding Medical Students Research Project" modeled on similar programs in western nations [6-8]. To understand students' career inclinations, we carried out this survey.

## Methods

All first-, second-, and third-year students participating in SUMC's "Outstanding Medical Students Research Project" were invited to participate in a survey on a volunteer basis. In the questionnaire (see Additional file 1), the survey was initially conducted to characterize student factors (grades, major course of study, and housing situation), family background (parents' professions, monthly monetary allowances), laboratory skills, scientific research interest, English language skills, computer skills, career aspirations, which we considered would affect the students' choice of career. Eighty-five students were assembled in a classroom at the beginning of the "Outstanding Medical Students Research Project" in summer 2007. After oral informed consent was obtained from these students, the six authors handed out the questionnaires and then collected them after about 20 minutes. The response rate was 100%. All data were entered into an electronic file and analyzed using Microsoft EXCEL statistical software (version: Microsoft Office Excel 2003 for Windows). All investigation procedures were approved by Shantou University Medical College Ethics Committee.

## Results

### Career aspirations of Chinese medical students

**Figure 1:**
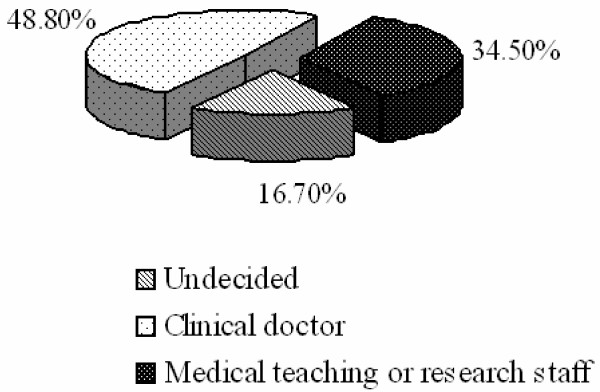
Career aspirations of medical students.

Career aspirations of medical students were categorized as: 1) medical teaching or research staff; 2) clinical doctors; or 3) undecided. Figure 1 shows that 35% of students reported "medical teaching or research staff", 48% reported "clinical doctor", and 17% were undecided. The proportion of males to females who reported "medical teaching or research staff" was 2:1, which is consistent with the proportion of males to females in this study sample.

### Family background

**Figure 2:**
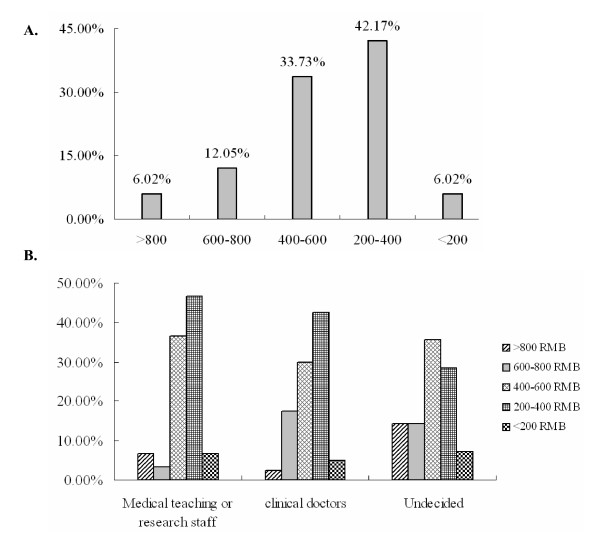
**Financial conditions of medical students.** A. Monthly monetary allowance from family. B. Student monthly allowance by career aspiration.

The mean reported monthly expenses were 439.55 RMB. Monthly allowances were categorized as: greater than 800 RMB, 600 to 800 RMB, 400 to 600 RMB, 200 to 400 RMB, or less than 200 RMB. Figure 2A shows that more than 50% of students received 400 RMB or more per month. Considering the average income in Southeast China, these students had above average income levels [9]. Analysis of allowance by career aspiration revealed no significant differences (Figure 2B). More than half of the students were children of farmers, fishermen or workers with modest incomes. However, these students did not differ from their peers in the level of financial support from family.

### Laboratory skills

Student experimental skills were assessed through a self-evaluation questionnaire in which students categorized themselves as: 1) excellent, with a high success rate in previous experiments; 2) good, with an average success rate; or 3) poor, with a low success rate.

**Figure 3:**
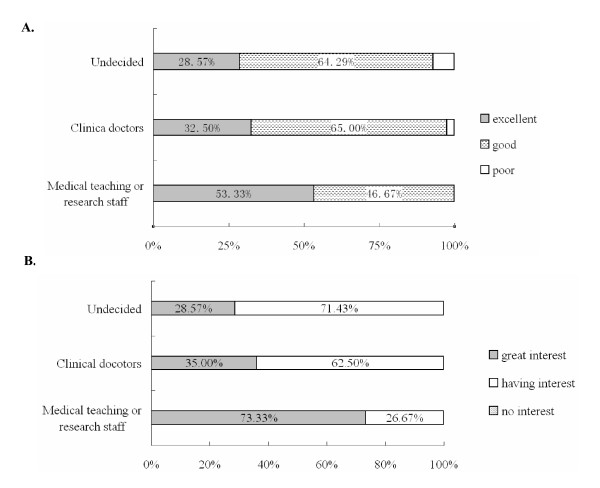
**Scientific characteristics of the students.** A. Student interest in basic science by career aspirations. B. Students' laboratory skill classified by career aspiration.

Figure 3A shows that 53.33% of students who chose "teaching or research" believe they have excellent experimental skills. Only 32.50% of the students choosing "clinical doctor" reported excellent laboratory skills. Undecided students reported the poorest laboratory skills.

### Scientific research interest

Students were asked to rate their interest in basic science as: 1) great interest; 2) some interest; or 3) no interest. All students reported at least some interest. As shown in Figure 3B, great interest was reported by 73.33% of those with teaching and research aspirations, 35% of those with clinical doctor aspirations, and 28.57% of those who were undecided.

### English language skills

**Figure 4:**
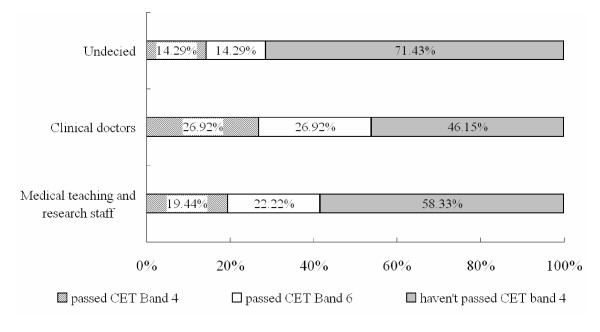
English skill level for medical students, as assessed by the College English Test.

The College English Test (CET) is used to evaluate English proficiency among Chinese college students. CET Band 4 is required for undergraduates and CET Band 6 for postgraduates. As indicated in Figure 4, half of the surveyed students had not yet passed the CET at the Band 4 level. This lack of proficiency was highest (71.43%) for the undecided group.

### Computer skills

**Figure 5:**
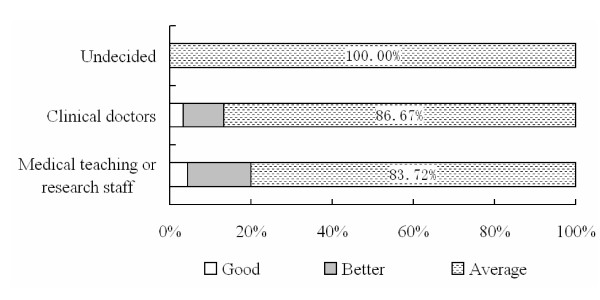
Student computer skills classified by career aspiration.

Student computer skill was categorized as 1) Good, capable of writing a simple program; 2) better, capable of designing a website, or 3) average. Figure 5 demonstrates poor overall computer skills among students; results were especially poor for the undecided group.

## Discussion and conclusions

### Changes in career aspirations

China's economic boom has impacted all aspects of Chinese society, including its healthcare system, which has shifted to market-driven services. Most of doctors in China have a higher average level of income. Figure 1 shows that 48% of students expressed intentions to become a "clinical doctor", and most undergraduates that we surveyed intend to find a job based on salary level. This result parallels the findings of Newton *et al. *[10] who reported income as being one of the most important factors in shaping the career choice of medical students in the United States. The traditional Chinese medical undergraduate pursues a career in clinical practice. However, our study demonstrated that a significant minority of students are now interested in nontraditional careers such as being medical teachers or researchers. Similar evaluations of medical students in western nations showed that the students often participate in scientific research in the form of a scholarly thesis or undergraduate research project [11-13]. Conversely, students in developing countries face funding constraints and limited scientific research training at their institutions [14].

Diverse career aspirations may be related to several trends in China. Doctors enjoy a relatively high income and elevated social status in China. Although researchers may have less stable incomes, their skills are highly sought and transferable to other areas, including the pharmaceutical industry, the biotechnology industry, the agricultural industry, private research institutes, and government research laboratories [15]. Another explanation of changing career aspirations may be doctors' dissatisfaction with current clinical practice and declining income levels. In China, there exists an unbalanced distribution of medical workers between cities and rural areas. The influx of doctors to cities has increased competition there for medical positions, decreasing the doctors' social status and value. Lim *et al. *[16] reported that Chinese doctors have a low rate of satisfaction about income level, career skills, and the medical care system generally. Research careers may be attractive to these dissatisfied doctors.

Careers in research and teaching may also appear more attractive because of the possibility for international research collaboration. China is increasingly benefiting from globalization and the opportunities in scientific research afforded by other countries. Therefore, increased emphasis on research activities may influence students' career aspirations [17,18]. SUMC has created the "Outstanding Medical Students Research Project" to support students interested in research, and such projects are important to encourage students to pursue research careers [19]. SUMC also embraces an international vision of research by inviting world-famous experts to give seminars, and by creating an environment conducive to international research collaboration [20]. SUMC is located in Guangdong Province, which is one of most prosperous areas in southern China. The employment conditions in this region are always a concern of the graduates. A limitation of this study is that we surveyed a small, select sample of students. Future studies will include a larger student sample, for example, the entire student body of SUMC, or even students from other medical schools in southern China. Such results would be more representative.

### Effects of personal factors

Student research interest plays a significant role in career aspiration [21]. The present study showed that 100% of students indicating a career aspiration in teaching or research had interest in basic research. This suggests that medical educators should work to stimulate student research interest at an early stage to maximize chances for a research career. Students with good laboratory skills, English proficiency and good computer skills are less likely to be undecided in their career aspirations. Schools should emphasize these skills to improve career opportunities for Chinese medical students.

### Guidance regarding career opportunities

Most of the undergraduates at SUMC are enrolled in a seven-year program that combines bachelor and master degree programs to produce Chinese doctors who are trained in clinical and research skills. The "Outstanding Medical Students Research Project" is designed to cultivate an interest in research among medical undergraduates. The program has been well-received thus far, and an increasing number of medical students have availed themselves of the opportunity to acquire basic research skills, which will meet the demand of their diversities on career choice.

## Competing interests

The authors declare that they have no competing interests.

## Authors' contributions

EL contributed to the conception and design of this study and data acquisition. All authors participated in the analysis or interpretation of data and the writing of this paper. All authors read and approved of the final manuscript.

## Pre-publication history

The pre-publication history for this paper can be accessed here:



## Supplementary Material

Additional file 1 **A survey of the factors affecting the career aspiration of undergraduate medical students.** A questionnaire used to investigate how some factors contribute to the determinants of career aspirations of medical students in southern China. Click here for file
