# Supplementary material for: Determinants of career aspirations of medical students in southern China
Source: BMC Med Educ. 2008 Dec 11;8:59. doi: 10.1186/1472-6920-8-59 (PMC2621218; doi:10.1186/1472-6920-8-59)
Supplement: Additional file 1 — A survey of the factors affecting the career aspiration of undergraduate medical students. A questionnaire used to investigate how some factors contribute to the determinants of career aspirations of medical students in southern China. [file 1472-6920-8-59-S1.doc]

| **A survey of the factors affecting the career aspiration**  **of undergraduate medical students** | | | | |
| --- | --- | --- | --- | --- |
| PART I: Background information | | | | |
| Name | | | | |
| Major course | | Year level: | | |
|  |  | Program: | | |
| PART Ⅱ: Family background | | | | |
| Parents professions | Low income:  A. Peasants; B. Fishermen, C. Laborers | | | High income:  A. Businessmen; B. Doctors; C. Teachers |
| Monthly monetary allowance | | | A. 800RMB  B. 600 to 800 RMB  C. 400 to 600 RMB  D. 200 to 400 RMB  E. less than 200 RMB | |
| PART Ⅲ: Characteristic related to Career choose | | | | |
| Career aspirations | | 1. Clinical doctor 2. Medical teaching or research staff 3. Undecided | | |
| English skills | | A. Passed CET – 6  B. Passed CET – 4  C. not passed CET | | |
| Computer skills | | A. Very good, capable of compiling a simple program  B. Better, capable of designing a website  C. Average, only able to use Microsoft Word etc. and browse the web | | |
| Laboratory skills | | A. Perform excellent, with a high success rate in previous experiments  B. Perform good, with an average success rate  C. Perform poor, with a low success rate | | |
| Scientific research interest | | A. With great interest, consider it as future career  B. With some interest, may be a career  C. No interest, cannot be my choose | | |
| Notes: 1. Translated from the origin Chinese version.  2. The information would not be open publicly. | | | | |
